# Supplementary material for: First characterisation of antimicrobial susceptibility and resistance of Neisseria gonorrhoeae isolates in Qatar, 2017–2020
Source: PLoS One. 2022 Mar 2;17(3):e0264737. doi: 10.1371/journal.pone.0264737 (PMC8890659; doi:10.1371/journal.pone.0264737)
Supplement: S2 Table — (DOCX) [file pone.0264737.s002.docx]

**Table S2** Patient characteristics and antimicrobial susceptibility in *Neisseria gonorrhoeae* isolates (n=433) from Qatar, 2017-2020

| **Isolate** | **Year** | **Sex** | **Age**  **(years)** | **Benzylpenicillin** | | **Ceftriaxone** | | **Ciprofloxacin** | | **Tetracycline** | | **Azithromycin** | |
| --- | --- | --- | --- | --- | --- | --- | --- | --- | --- | --- | --- | --- | --- |
|  |  |  |  | **MIC** | **Result** | **MIC** | **Result** | **MIC** | **Result** | **MIC** | **Result** | **MIC** | **Result** |
| 1 | 2017 | M | 24 | 1 | I | 0.032 | S | 8 | R | - | - | - | - |
| 2 | 2017 | M | 48 | 0.38 | I | 0.012 | S | 0.38 | I | - | - | - | - |
| 3 | 2017 | M | 40 | 0.032 | S | 0.002 | S | 16 | R | 0.19 | S | - | - |
| 4 | 2017 | M | 20 | 1.5 | R | 0.032 | S | 4 | R | 4 | R | - | - |
| 5 | 2017 | M | 21 | 0.094 | I | 0.003 | S | 4 | R | - | - | - | - |
| 6 | 2017 | M | 21 | 256 | R | 0.006 | S | 2 | R | 3 | R | - | - |
| 7 | 2017 | M | 36 | 8 | R | 0.008 | S | 32 | R | - | - | - | - |
| 8 | 2017 | M | 25 | 0.064 | S | 0.004 | S | 4 | R | 1.5 | R | - | - |
| 9 | 2017 | M | 27 | 0.25 | I | 0.004 | S | 0.75 | R | 24 | R | - | - |
| 10 | 2017 | M | 30 | 0.25 | I | 0.003 | S | 8 | R | 24 | R | - | - |
| 11 | 2017 | F | 30 | 1 | I | 0.003 | S | 0.5 | I | 16 | R | - | - |
| 12 | 2017 | M | 24 | 0.75 | I | 0.023 | S | 8 | R | - | - | - | - |
| 13 | 2017 | M | 31 | 1.5 | R | 0.012 | S | 32 | R | 3 | R | - | - |
| 14 | 2017 | M | 30 | 0.38 | I | 0.023 | S | 1.5 | R | 1.5 | R | - | - |
| 15 | 2017 | M | 33 | 12 | R | 0.064 | S | 1 | R | - | - | - | - |
| 16 | 2017 | M | 21 | 0.75 | I | 0.023 | S | 8 | R | - | - | - | - |
| 17 | 2017 | M | 16 | 16 | R | 0.004 | S | 1 | R | - | - | - | - |
| 18 | 2017 | M | 25 | 12 | R | 0.003 | S | 1.5 | R | - | - | - | - |
| 19 | 2017 | M | 20 | 0.5 | I | 0.012 | S | 6 | R | - | - | - | - |
| 20 | 2017 | M | 17 | 8 | R | 0.002 | S | 2 | R | - | - | - | - |
| 21 | 2017 | M | 26 | 0.25 | I | 0.006 | S | 2 | R | - | - | - | - |
| 22 | 2017 | M | 21 | 0.125 | I | 0.012 | S | 3 | R | - | - | - | - |
| 23 | 2017 | M | 28 | 0.19 | I | 0.032 | S | 8 | R | - | - | - | - |
| 24 | 2017 | M | 25 | 0.75 | I | 0.002 | S | 8 | R | - | - | - | - |
| 25 | 2017 | M | 31 | 0.016 | S | 0.002 | S | 2 | R | - | - | - | - |
| 26 | 2017 | M | 27 | 0.125 | I | 0.004 | S | 0.006 | S | - | - | - | - |
| 27 | 2017 | M | 21 | 0.19 | I | 0.012 | S | 4 | R | - | - | - | - |
| 28 | 2017 | M | 26 | 0.38 | I | 0.008 | S | 2 | R | - | - | - | - |
| 29 | 2017 | M | 36 | 32 | R | 0.004 | S | 1 | R | - | - | - | - |
| 30 | 2017 | M | 24 | 8 | R | 0.006 | S | 1.5 | R | - | - | - | - |
| 31 | 2017 | M | 22 | 0.064 | S | 0.006 | S | 32 | R | - | - | - | - |
| 32 | 2017 | M | 24 | 0.75 | I | 0.012 | S | 6 | R | 1.5 | R | - | - |
| 33 | 2017 | M | 27 | 6 | R | 0.004 | S | 1.5 | R | 16 | R | - | - |
| 34 | 2017 | F | 29 | 4 | R | 0.003 | S | 1 | R | 16 | R | - | - |
| 35 | 2017 | F | 50 | 0.19 | I | 0.023 | S | 3 | R | 32 | R | - | - |
| 36 | 2017 | M | 25 | 0.38 | I | 0.016 | S | 32 | R | - | - | - | - |
| 37 | 2017 | M | 16 | 12 | R | 0.003 | S | 1 | R | 1 | I | - | - |
| 38 | 2017 | M | 25 | 0.5 | I | 0.012 | S | 32 | R | 2 | R | - | - |
| 39 | 2017 | M | 29 | 0.125 | I | 0.002 | S | 4 | R | 1.5 | R | - | - |
| 40 | 2017 | M | 16 | 3 | R | 0.002 | S | 1.5 | R | 8 | R | - | - |
| 41 | 2017 | M | 29 | 32 | R | -^*^ | S^*^ | 4 | R | 0.5 | I | - | - |
| 42 | 2017 | M | 30 | 0.016 | S | 0.002 | S | - | - | 16 | R | - | - |
| 43 | 2017 | M | 22 | 0.064 | S | 0.003 | S | 0.002 | S | 0.25 | S | - | - |
| 44 | 2017 | M | 39 | 192 | R | 0.003 | S | 2 | R | 24 | R | - | - |
| 45 | 2017 | M | 21 | 3 | R | 0.003 | S | 1.5 | R | 12 | R | - | - |
| 46 | 2017 | M | 29 | 0.016 | S | 0.002 | S | 0.016 | S | 1 | I | - | - |
| 47 | 2017 | M | 23 | 16 | R | 0.003 | S | 3 | R | 6 | R | - | - |
| 48 | 2017 | M | 24 | 0.016 | S | 0.003 | S | 32 | R | 0.25 | S | - | - |
| 49 | 2017 | M | 36 | 0.25 | I | 0.003 | S | 0.75 | R | 32 | R | - | - |
| 50 | 2017 | M | 16 | 0.38 | I | 0.003 | S | 0.004 | S | 0.5 | I | - | - |
| 51 | 2017 | M | 36 | 0.125 | I | 0.012 | S | 8 | R | 1 | I | - | - |
| 52 | 2017 | M | 44 | 0.5 | I | 0.023 | S | - | - | 32 | R | - | - |
| 53 | 2017 | M | 32 | 0.25 | I | 0.002 | S | - | - | 4 | R | - | - |
| 54 | 2017 | M | 27 | 0.38 | I | 0.023 | S | - | - | 1.5 | R | - | - |
| 55 | 2017 | M | 30 | 4 | R | 0.004 | S | - | - | 16 | R | - | - |
| 56 | 2017 | M | 18 | 0.25 | I | 0.012 | S | - | - | 1.5 | R | - | - |
| 57 | 2017 | M | 32 | 0.125 | I | 0.004 | S | - | - | 0.38 | I | - | - |
| 58 | 2017 | M | 18 | 0.25 | I | 0.023 | S | - | - | 1 | I | - | - |
| 59 | 2017 | M | 21 | 0.016 | S | 0.002 | S | - | - | 0.25 | S | - | - |
| 60 | 2017 | M | 21 | 96 | R | 0.003 | S | - | - | 16 | R | - | - |
| 61 | 2017 | M | 22 | 0.38 | I | 0.012 | S | - | - | 0.5 | I | - | - |
| 62 | 2017 | M | 24 | 1 | I | 0.19 | S | - | - | 3 | R | - | - |
| 63 | 2017 | M | 23 | 1 | I | 0.006 | S | 1 | R | 8 | R | - | - |
| 64 | 2017 | F | 29 | 0.25 | I | 0.006 | S | 2 | R | 0.25 | S | - | - |
| 65 | 2017 | M | 23 | 0.125 | I | 0.016 | S | 1 | R | 0.5 | I | - | - |
| 66 | 2017 | M | 24 | 0.047 | S | 0.002 | S | 0.125 | I | 2 | R | - | - |
| 67 | 2017 | M | 23 | 0.75 | I | 0.25 | S | 0.047 | S | 0.064 | S | - | - |
| 68 | 2017 | M | 21 | 0.5 | I | 0.032 | S | 6 | R | 0.75 | I | - | - |
| 69 | 2017 | M | 18 | 16 | R | 0.032 | S | 8 | R | 12 | R | - | - |
| 70 | 2017 | M | 31 | 1 | I | 0.032 | S | 6 | R | 2 | R | - | - |
| 71 | 2017 | M | 28 | 0.016 | S | 0.002 | S | 4 | R | 0.38 | I | - | - |
| 72 | 2017 | M | 26 | 2 | R | 0.003 | S | 8 | R | - | - | - | - |
| 73 | 2017 | M | 17 | 0.125 | I | 0.002 | S | 1 | R | 12 | R | - | - |
| 74 | 2017 | M | 33 | 0.125 | I | 0.003 | S | 0.5 | I | - | - | - | - |
| 75 | 2017 | M | 17 | 8 | R | 0.004 | S | 0.75 | R | 16 | R | - | - |
| 76 | 2017 | M | 19 | 2 | R | 0.003 | S | 3 | R | - | - | - | - |
| 77 | 2017 | M | 28 | 0.25 | I | 0.016 | S | 2 | R | - | - | - | - |
| 78 | 2017 | M | 18 | 0.38 | I | 0.016 | S | 2 | R | 1 | I | - | - |
| 79 | 2017 | M | 21 | 4 | R | 0.004 | S | - | - | 32 | R | - | - |
| 80 | 2017 | M | 27 | 0.064 | S | 0.003 | S | 0.75 | R | - | - | - | - |
| 81 | 2017 | M | 18 | 0.125 | I | 0.004 | S | 0.75 | R | 16 | R | - | - |
| 82 | 2017 | M | 23 | 4 | R | 0.004 | S | - | - | 24 | R | - | - |
| 83 | 2017 | M | 27 | 8 | R | 0.003 | S | - | - | 12 | R | - | - |
| 84 | 2017 | F | 60 | 0.25 | I | 0.003 | S | 0.002 | S | 16 | R | - | - |
| 85 | 2017 | M | 37 | 0.064 | S | 0.003 | S | 0.003 | S | 0.5 | I | - | - |
| 86 | 2017 | M | 21 | 3 | R | 0.003 | S | 0.5 | I | 12 | R | - | - |
| 87 | 2017 | M | 21 | 2 | R | 0.008 | S | 1 | R | 16 | R | - | - |
| 88 | 2017 | M | 19 | 0.5 | I | 0.047 | S | 2 | R | 1 | I | - | - |
| 89 | 2017 | M | 20 | 0.5 | I | 0.047 | S | 4 | R | 1.5 | R | - | - |
| 90 | 2017 | M | 29 | 0.125 | I | 0.003 | S | 0.75 | R | 16 | R | - | - |
| 91 | 2017 | M | 26 | 0.094 | I | 0.002 | S | 0.75 | R | 16 | R | - | - |
| 92 | 2017 | M | 19 | 6 | R | 0.004 | S | 0.75 | R | 2 | R | - | - |
| 93 | 2017 | M | 26 | 0.125 | I | 0.002 | S | 0.75 | R | 0.5 | I | - | - |
| 94 | 2017 | M | 18 | 256 | R | 0.004 | S | 0.75 | R | 16 | R | - | - |
| 95 | 2017 | M | 19 | 0.38 | I | 0.032 | S | 8 | R | 1 | I | - | - |
| 96 | 2017 | M | 34 | 4 | R | 0.002 | S | 0.75 | R | 12 | R | - | - |
| 97 | 2017 | M | 18 | 0.064 | S | 0.008 | S | 0.38 | I | 16 | R | - | - |
| 98 | 2017 | M | 19 | 2 | R | 0.002 | S | 0.38 | I | 1.5 | R | - | - |
| 99 | 2017 | M | 21 | 4 | R | 0.003 | S | 0.38 | I | 6 | R | - | - |
| 100 | 2017 | M | 26 | 0.125 | I | 0.004 | S | 0.38 | I | 16 | R | - | - |
| 101 | 2017 | M | 16 | 0.75 | I | 0.047 | S | 4 | R | 1.5 | R | - | - |
| 102 | 2017 | M | 21 | 8 | R | 0.004 | S | 32 | R | 16 | R | - | - |
| 103 | 2017 | M | 19 | 0.125 | I | 0.008 | S | 0.38 | I | 0.25 | S | - | - |
| 104 | 2017 | M | 19 | 12 | R | 0.002 | S | 0.25 | I | 12 | R | - | - |
| 105 | 2017 | M | 20 | 0.38 | I | 0.008 | S | 1.5 | R | 0.75 | I | - | - |
| 106 | 2017 | M | 24 | 8 | R | 0.125 | S | 0.38 | I | 12 | R | - | - |
| 107 | 2017 | M | 19 | 8 | R | 0.003 | S | 0.38 | I | 24 | R | - | - |
| 108 | 2017 | M | 19 | 0.5 | I | 0.023 | S | 3 | R | 1.5 | R | - | - |
| 109 | 2017 | M | 18 | 2 | R | 0.023 | S | 2 | R | 2 | R | - | - |
| 110 | 2018 | M | 30 | 4 | R | 0.002 | S | 0.012 | S | 8 | R | - | - |
| 111 | 2018 | M | 25 | - | - | 0.003 | S | 0.38 | I | 3 | R | - | - |
| 112 | 2018 | M | 24 | 6 | R | 0.003 | S | 0.75 | R | 8 | R | - | - |
| 113 | 2018 | M | 32 | 4 | R | 0.002 | S | 0.38 | I | 0.75 | I | - | - |
| 114 | 2018 | M | 17 | 0.38 | I | 0.012 | S | 1 | R | 0.5 | I | - | - |
| 115 | 2018 | M | 18 | 0.19 | I | 0.002 | S | 0.002 | S | 0.5 | I | - | - |
| 116 | 2018 | M | 28 | 0.38 | I | 0.023 | S | 1.5 | R | 0.75 | I | - | - |
| 117 | 2018 | M | 21 | 0.38 | I | 0.032 | S | 32 | R | 1 | I | - | - |
| 118 | 2018 | M | 23 | 0.19 | I | 0.016 | S | 2 | R | 0.75 | I | - | - |
| 119 | 2018 | M | 19 | 0.094 | I | 0.002 | S | 1 | R | 12 | R | - | - |
| 120 | 2018 | M | 31 | 0.38 | I | 0.016 | S | 1 | R | 0.75 | I | - | - |
| 121 | 2018 | M | 20 | 0.38 | I | 0.006 | S | 0.25 | I | 6 | R | - | - |
| 122 | 2018 | M | 17 | 24 | R | 0.004 | S | 0.5 | I | 12 | R | - | - |
| 123 | 2018 | M | 17 | 8 | R | 0.004 | S | 0.5 | I | 16 | R | - | - |
| 124 | 2018 | M | 18 | - | - | 0.008 | S | 1.5 | R | 0.25 | S | - | - |
| 125 | 2018 | M | 30 | - | - | 0.002 | S | 0.002 | S | 6 | R | - | - |
| 126 | 2018 | M | 33 | 2 | R | 0.002 | S | 0.19 | I | 0.125 | S | - | - |
| 127 | 2018 | M | 24 | 0.38 | I | 0.008 | S | 0.75 | R | 0.25 | S | - | - |
| 128 | 2018 | M | 33 | 4 | R | 0.002 | S | 0.75 | R | 6 | R | - | - |
| 129 | 2018 | M | 21 | 0.75 | I | 0.032 | S | 4 | R | 1 | I | - | - |
| 130 | 2018 | M | 49 | - | - | 0.002 | S | 1.5 | R | 0.38 | I | - | - |
| 131 | 2018 | M | 31 | 0.38 | I | 0.016 | S | 2 | R | 1.5 | R | - | - |
| 132 | 2018 | M | 17 | 0.38 | I | 0.003 | S | 12 | R | 0.5 | I | - | - |
| 133 | 2018 | M | 25 | 0.5 | I | 0.016 | S | 4 | R | 1 | I | - | - |
| 134 | 2018 | M | 19 | - | - | 0.003 | S | 0.002 | S | 16 | R | - | - |
| 135 | 2018 | M | 21 | 0.75 | I | 0.023 | S | 2 | R | 1 | I | - | - |
| 136 | 2018 | M | 28 | 0.064 | S | 0.003 | S | 0.5 | I | 8 | R | - | - |
| 137 | 2018 | M | 19 | 16 | R | 0.002 | S | 1 | R | 12 | R | - | - |
| 138 | 2018 | M | 21 | - | - | 0.002 | S | 0.38 | I | 16 | R | - | - |
| 139 | 2018 | M | 31 | 256 | R | 0.003 | S | 0.19 | I | 8 | R | - | - |
| 140 | 2018 | M | 21 | 192 | R | 0.002 | S | 1.5 | R | 16 | R | - | - |
| 141 | 2018 | M | 31 | 0.5 | I | 0.023 | S | 2 | R | 0.75 | I | - | - |
| 142 | 2018 | M | 28 | 0.25 | I | 0.006 | S | 2 | R | 0.75 | I | - | - |
| 143 | 2018 | M | 27 | 0.094 | I | 0.002 | S | 0.002 | S | 24 | R | - | - |
| 144 | 2018 | M | 19 | 0.38 | I | 0.008 | S | 0.125 | I | 16 | R | - | - |
| 145 | 2018 | M | 16 | 0.38 | I | 0.003 | S | 8 | R | 0.75 | I | - | - |
| 146 | 2018 | M | 29 | 0.19 | I | 0.012 | S | 4 | R | 0.75 | I | - | - |
| 147 | 2018 | M | 17 | 16 | R | 0.002 | S | 0.38 | I | 12 | R | - | - |
| 148 | 2018 | M | 17 | 32 | R | 0.003 | S | 2 | R | 16 | R | - | - |
| 149 | 2018 | M | 45 | 3 | R | 0.006 | S | 2 | R | 24 | R | - | - |
| 150 | 2018 | M | 41 | 0.016 | S | 0.002 | S | 3 | R | 0.19 | S | - | - |
| 151 | 2018 | M | 34 | 0.016 | S | 0.002 | S | 4 | R | 0.25 | S | - | - |
| 152 | 2018 | M | 17 | 0.25 | I | 0.008 | S | 2 | R | 0.5 | I | - | - |
| 153 | 2018 | M | 20 | 0.016 | S | 0.002 | S | 0.002 | S | 0.19 | S | - | - |
| 154 | 2018 | M | 27 | 0.047 | S | 0.002 | S | 0.5 | I | 3 | R | - | - |
| 155 | 2018 | M | 19 | 0.5 | I | 0.012 | S | 3 | R | 0.5 | I | - | - |
| 156 | 2018 | M | 25 | 16 | R | 0.003 | S | 1 | R | 0.75 | I | - | - |
| 157 | 2018 | M | 22 | 0.094 | I | 0.003 | S | 0.5 | I | 8 | R | - | - |
| 158 | 2018 | M | 31 | 0.19 | I | 0.012 | S | 4 | R | 1 | I | - | - |
| 159 | 2018 | M | 30 | 1 | I | 0.032 | S | 4 | R | 1.5 | R | - | - |
| 160 | 2018 | M | 28 | 0.5 | I | 0.023 | S | 6 | R | 1 | I | - | - |
| 161 | 2018 | M | 28 | 0.19 | I | 0.012 | S | 1 | R | 0.75 | I | - | - |
| 162 | 2018 | M | 40 | 0.19 | I | 0.003 | S | 0.75 | R | 32 | R | - | - |
| 163 | 2018 | M | 31 | 16 | R | 0.002 | S | 0.5 | I | - | - | - | - |
| 164 | 2018 | M | 25 | 0.75 | I | 0.012 | S | 2 | R | - | - | - | - |
| 165 | 2018 | M | 25 | 1 | I | 0.064 | S | 4 | R | - | - | - | - |
| 166 | 2018 | M | 21 | 0.19 | I | 0.003 | S | - | - | 0.38 | I | - | - |
| 167 | 2018 | M | 31 | 1.5 | R | 0.008 | S | - | - | 1.5 | R | - | - |
| 168 | 2018 | M | 23 | 256 | R | 0.004 | S | 2 | R | 2 | R | - | - |
| 169 | 2018 | M | 31 | 0.064 | S | 0.002 | S | 0.19 | I | 0.25 | S | - | - |
| 170 | 2018 | M | 34 | 0.38 | I | 0.003 | S | 0.25 | I | 8 | R | - | - |
| 171 | 2018 | M | 26 | 2 | R | 0.047 | S | 12 | R | 2 | R | - | - |
| 172 | 2018 | M | 19 | 0.25 | I | 0.016 | S | 33 | R | 1 | I | - | - |
| 173 | 2018 | M | 17 | 3 | R | 0.003 | S | 1.5 | R | - | - | - | - |
| 174 | 2018 | M | 20 | 0.25 | I | 0.006 | S | 1 | R | - | - | - | - |
| 175 | 2018 | M | 18 | 0.5 | I | 0.016 | S | 6 | R | - | - | - | - |
| 176 | 2018 | M | 30 | 0.094 | I | 0.002 | S | - | - | 4 | R | - | - |
| 177 | 2018 | M | 30 | 2 | R | - | - | 4 | R | 0.094 | S | - | - |
| 178 | 2018 | M | 18 | 0.19 | I | 0.004 | S | 0.75 | R | 24 | R | - | - |
| 179 | 2018 | M | 17 | 0.5 | I | 0.023 | S | 6 | R | 32 | R | - | - |
| 180 | 2018 | M | 18 | 0.19 | I | 0.004 | S | 0.75 | R | 24 | R | - | - |
| 181 | 2018 | M | 16 | 0.5 | I | 0.023 | S | 6 | R | 32 | R | - | - |
| 182 | 2018 | M | 18 | 0.38 | I | 0.008 | S | - | - | 0.75 | I | 0.25 | S |
| 183 | 2018 | M | 41 | 0.125 | I | 0.003 | S | 32 | R | 0.75 | I | - | - |
| 184 | 2018 | M | 21 | 0.125 | I | 0.002 | S | 0.002 | S | 24 | R | - | - |
| 185 | 2018 | M | 23 | 0.064 | S | 0.002 | S | 0.75 | R | - | - | - | - |
| 186 | 2018 | M | 13 | 0.125 | I | 0.003 | S | 0.002 | S | 16 | R | 0.19 | S |
| 187 | 2018 | M | 37 | 0.064 | S | 0.006 | S | 2 | R | - | - | 0.047 | S |
| 188 | 2018 | M | 27 | 0.75 | I | 0.006 | S | 0.75 | R | 0.25 | S | 2 | R |
| 189 | 2018 | F | 30 | 0.125 | I | 0.002 | S | 4 | R | 0.5 | I | - | - |
| 190 | 2018 | M | 16 | 1.5 | R | 0.023 | S | 4 | R | 1.5 | R | 0.75 | S |
| 191 | 2018 | M | 25 | 3 | R | 0.002 | S | 0.25 | I | 4 | R | 0.032 | S |
| 192 | 2018 | M | 16 | 0.016 | S | 0.002 | S | 0.5 | I | 2 | R | 0.125 | S |
| 193 | 2018 | F | 39 | 0.094 | I | 0.003 | S | 3 | R | 0.125 | S | 0.094 | S |
| 194 | 2018 | M | 17 | 0.064 | S | 0.002 | S | 0.004 | S | 0.19 | S | 0.25 | S |
| 195 | 2018 | M | 19 | - | - | 0.003 | S | 0.5 | I | 8 | R | 0.064 | S |
| 196 | 2018 | M | 31 | - | - | 0.002 | S | 1.5 | R | 16 | R | 0.19 | S |
| 197 | 2018 | M | 56 | 0.094 | I | 0.003 | S | 0.75 | R | 0.5 | I | 0.032 | S |
| 198 | 2018 | M | 22 | 0.19 | I | 0.004 | S | 0.002 | S | 16 | R | 0.25 | S |
| 199 | 2018 | M | 26 | 1.5 | R | 0.016 | S | 0.5 | I | 8 | R | 0.75 | S |
| 200 | 2018 | M | 22 | 3 | R | 0.002 | S | 0.75 | R | 0.5 | I | 0.016 | S |
| 201 | 2018 | M | 57 | - | - | 0.094 | S | 8 | R | 1.5 | R | 1 | S |
| 202 | 2018 | M | 22 | - | - | 0.003 | S | 0.75 | R | 12 | R | 0.064 | S |
| 203 | 2018 | M | 22 | 0.064 | S | 0.008 | S | 0.5 | I | 12 | R | 0.047 | S |
| 204 | 2018 | M | 21 | - | - | 0.032 | S | 4 | R | 1 | I | 0.5 | S |
| 205 | 2018 | M | 19 | - | - | 0.006 | S | 4 | R | 0.75 | I | 0.5 | S |
| 206 | 2018 | M | 31 | 0.125 | I | 0.002 | S | 2 | R | 0.19 | S | 0.125 | S |
| 207 | 2018 | M | 59 | - | - | 0.032 | S | 4 | R | 0.125 | S | 1 | S |
| 208 | 2018 | M | 15 | 0.5 | I | 0.003 | S | 0.38 | I | 24 | R | 0.094 | S |
| 209 | 2018 | M | 42 | 0.75 | I | 0.008 | S | 3 | R | 0.5 | I | 0.75 | S |
| 210 | 2018 | M | 19 | - | - | 0.004 | S | 1.5 | R | 0.75 | I | 0.023 | S |
| 211 | 2018 | M | 21 | 0.38 | I | 0.008^*^ | S^*^ | 0.094 | I | 0.064 | S | 2 | R |
| 212 | 2018 | M | 23 | 0.75 | I | 0.023 | S | 2 | R | 0.75 | I | 0.75 | S |
| 213 | 2018 | M | 24 | 0.094 | I | 0.002 | S | 0.003 | S | 0.38 | I | 0.125 | S |
| 214 | 2018 | M | 43 | 2 | R | 0.016 | S | 0.125 | I | 16 | R | 0.19 | S |
| 215 | 2018 | M | 30 | 0.047 | S | 0.002 | S | 0.25 | I | 2 | R | 0.016 | S |
| 216 | 2018 | M | 19 | 0.016 | S | 0.002 | S | 0.002 | S | 0.19 | S | 0.064 | S |
| 217 | 2018 | M | 21 | 0.38 | I | 0.023 | S | - | - | 1 | I | - | - |
| 218 | 2018 | M | 24 | 0.064 | S | 0.002 | S | 0.5 | I | 12 | R | 0.75 | S |
| 219 | 2018 | M | 20 | 0.125 | I | 0.008 | S | 1.5 | R | 0.38 | I | 0.25 | S |
| 220 | 2018 | M | 43 | 6 | R | 0.003 | S | 0.75 | R | 0.38 | I | 0.064 | S |
| 221 | 2018 | M | 19 | 0.094 | I | 0.002 | S | 1 | R | 8 | R | 0.032 | S |
| 222 | 2018 | M | 22 | 0.19 | I | 0.006 | S | 0.75 | R | 0.25 | S | 0.19 | S |
| 223 | 2018 | M | 24 | 256 | R | 0.003 | S | 6 | R | 16 | R | 0.023 | S |
| 224 | 2018 | M | 18 | 0.75 | I | 0.016 | S | 0.125 | I | 0.75 | I | 0.5 | S |
| 225 | 2018 | M | 31 | 0.094 | I | 0.002 | S | 1.5 | R | 0.5 | I | 0.25 | S |
| 226 | 2018 | M | 19 | 1 | I | 0.064 | S | 0.75 | R | 12 | R | 0.19 | S |
| 227 | 2018 | M | 21 | 0.5 | I | 0.002 | S | 0.19 | I | 0.38 | I | 0.032 | S |
| 228 | 2018 | M | 27 | 0.064 | S | 0.003 | S | 1.5 | R | 0.25 | S | - | - |
| 229 | 2018 | M | 43 | 0.19 | I | 0.003 | S | 1.5 | R | 12 | R | 0.094 | S |
| 230 | 2018 | M | 24 | 0.19 | I | 0.012 | S | 0.125 | I | 0.19 | S | 0.19 | S |
| 231 | 2018 | M | 18 | 0.19 | I | 0.023 | S | 1.5 | R | 0.19 | S | 0.25 | S |
| 232 | 2018 | M | 25 | 0.25 | I | 0.008 | S | 1.5 | R | 0.19 | S | 0.25 | S |
| 233 | 2018 | M | 23 | 0.125 | I | 0.002 | S | 0.5 | I | 0.125 | S | 0.047 | S |
| 234 | 2018 | M | 47 | 32 | R | 0.006 | S | 1.5 | R | 16 | R | 0.094 | S |
| 235 | 2018 | M | 26 | 0.5 | I | 0.023 | S | 4 | R | 0.5 | I | 0.5 | S |
| 236 | 2018 | M | 22 | 0.125 | I | 0.003 | S | 0.38 | I | 8 | R | 0.023 | S |
| 237 | 2018 | M | 24 | - | - | 0.023 | S | 4 | R | 0.75 | I | 0.38 | S |
| 238 | 2018 | M | 30 | - | - | 0.003 | S | 1 | R | 12 | R | 0.047 | S |
| 239 | 2018 | M | 28 | - | - | 0.002 | S | 0.003 | S | 0.5 | I | 0.047 | S |
| 240 | 2018 | M | 77 | 0.125 | I | 0.006 | S | 6 | R | 0.38 | I | 0.19 | S |
| 241 | 2018 | M | 41 | 4 | R | 0.002 | S | 0.5 | I | 6 | R | 0.023 | S |
| 242 | 2018 | M | 30 | 16 | R | 0.002 | S | 0.5 | I | 6 | R | 0.047 | S |
| 243 | 2018 | M | 21 | 0.19 | I | 0.004 | S | 2 | R | 0.75 | I | 0.25 | S |
| 244 | 2018 | M | 36 | 0.125 | I | 0.006 | S | 3 | R | 0.75 | I | 0.125 | S |
| 245 | 2018 | M | 27 | 0.064 | S | 0.002 | S | 0.38 | I | 0.25 | S | 0.125 | S |
| 246 | 2018 | M | 18 | 4 | R | 0.002 | S | 3 | R | 4 | R | 0.19 | S |
| 247 | 2018 | M | 19 | 16 | R | 0.002 | S | 0.002 | S | 0.75 | I | 0.094 | S |
| 248 | 2018 | M | 27 | 1 | I | 0.002 | S | 0.5 | I | 16 | R | 0.19 | S |
| 249 | 2018 | M | 24 | 0.064 | S | 0.002 | S | 4 | R | 0.25 | S | 0.064 | S |
| 250 | 2018 | F | 42 | 24 | R | 0.003 | S | 0.75 | R | 12 | R | 0.094 | S |
| 251 | 2018 | M | 31 | 0.5 | I | 0.016 | S | 16 | R | 0.75 | I | 0.094 | S |
| 252 | 2018 | M | 38 | 0.094 | I | 0.012 | S | 12 | R | 0.25 | S | 0.094 | S |
| 253 | 2018 | M | 23 | 4 | R | 0.002 | S | 0.25 | I | 6 | R | 0.023 | S |
| 254 | 2018 | M | 40 | 0.75 | I | 0.003 | S | 0.064 | S | 0.75 | I | 0.094 | S |
| 255 | 2018 | M | 24 | 4 | R | 0.002 | S | 0.38 | I | 2 | R | 0.094 | S |
| 256 | 2018 | M | 20 | 0.5 | I | 0.012 | S | 8 | R | 0.5 | I | 0.38 | S |
| 257 | 2018 | M | 19 | 0.032 | S | 0.002 | S | 0.002 | S | 0.19 | S | 0.016 | S |
| 258 | 2018 | F | 60 | 1.5 | R | 0.002 | S | 0.5 | I | 3 | R | 0.125 | S |
| 259 | 2018 | M | 34 | 0.016 | S | 0.002 | S | 4 | R | 0.125 | S | 0.125 | S |
| 260 | 2018 | M | 23 | 0.19 | I | 0.008 | S | 3 | R | 0.5 | I | 0.094 | S |
| 261 | 2018 | M | 18 | 0.064 | S | 0.002 | S | 0.002 | S | 0.25 | S | 0.016 | S |
| 262 | 2019 | M | 23 | 4 | R | 0.016 | S | 0.38 | I | 0.125 | S | 0.047 | S |
| 263 | 2019 | M | 20 | - | - | 0.016 | S | 0.38 | I | 3 | R | 0.032 | S |
| 264 | 2019 | M | 18 | - | - | 0.016 | S | 0.25 | I | 4 | R | 0.064 | S |
| 265 | 2019 | M | 20 | 0.75 | I | 0.016 | S | 0.002 | S | 0.38 | I | 0.064 | S |
| 266 | 2019 | M | 20 | 0.38 | I | 0.016 | S | 3 | R | 0.75 | I | 0.5 | S |
| 267 | 2019 | M | 26 | 8 | R | 0.016 | S | 0.25 | I | 12 | R | 0.064 | S |
| 268 | 2019 | M | 22 | 0.19 | I | 0.016 | S | 0.004 | S | 0.5 | I | 1.5 | R |
| 269 | 2019 | M | 20 | 1 | I | 0.016 | S | 1 | R | 8 | R | 0.047 | S |
| 270 | 2019 | M | 20 | - | - | 0.016 | S | - | - | 4 | R | 0.094 | S |
| 271 | 2019 | F | 28 | 0.094 | I | 0.016 | S | 1 | R | 4 | R | 0.016 | S |
| 272 | 2019 | M | 26 | - | - | 0.023 | S | - | - | 1 | I | 0.25 | S |
| 273 | 2019 | M | 19 | - | - | 0.016 | S | - | - | 0.25 | S | 0.047 | S |
| 274 | 2019 | M | 39 | - | - | 0.023 | S | 4 | R | 0.5 | I | 0.25 | S |
| 275 | 2019 | M | 23 | - | - | 0.032 | S | - | - | 0.75 | I | 0.032 | S |
| 276 | 2019 | M | 18 | 0.047 | S | 0.016 | S | 0.002 | S | 12 | R | 0.125 | S |
| 277 | 2019 | M | 33 | - | - | 0.016 | S | - | - | 0.125 | S | 0.094 | S |
| 278 | 2019 | M | 24 | - | - | 0.016 | S | 16 | R | 8 | R | 0.032 | S |
| 279 | 2019 | M | 16 | 0.75 | I | 0.016 | S | 0.002 | S | 0.25 | S | 0.094 | S |
| 280 | 2019 | M | 28 | 0.094 | I | 0.016 | S | 1 | R | 1 | I | 0.023 | S |
| 281 | 2019 | M | 25 | - | - | 0.016 | S | - | - | 0.25 | S | 0.094 | S |
| 282 | 2019 | M | 35 | 2 | R | 0.016 | S | 0.38 | I | 0.19 | S | 0.064 | S |
| 283 | 2019 | M | 19 | 16 | R | 0.016 | S | 0.38 | I | 24 | R | 0.032 | S |
| 284 | 2019 | M | 25 | - | - | 0.016 | S | 1.5 | R | 6 | R | 0.047 | S |
| 285 | 2019 | M | 20 | - | - | 0.016 | S | - | - | 0.5 | I | 0.125 | S |
| 286 | 2019 | M | 26 | 0.125 | I | - | - | - | - | 8 | R | 0.016 | S |
| 287 | 2019 | M | 44 | 3 | R | - | - | - | - | 4 | R | 0.016 | S |
| 288 | 2019 | M | 26 | 0.75 | I | 0.023 | S | 0.023 | S | 0.5 | I | 0.064 | S |
| 289 | 2019 | M | 20 | 16 | R | 0.023 | S | 0.75 | R | 12 | R | 0.023 | S |
| 290 | 2019 | M | 22 | 0.25 | I | 0.008 | S | 16 | R | 0.75 | I | 0.25 | S |
| 291 | 2019 | M | 19 | 0.047 | S | 0.016 | S | 0.002 | S | 3 | R | 0.023 | S |
| 292 | 2019 | M | 21 | 0.064 | S | - | - | - | - | 0.25 | S | 0.016 | S |
| 293 | 2019 | M | 19 | 0.023 | S | - | - | - | - | 0.032 | S | 0.064 | S |
| 294 | 2019 | M | 18 | - | - | - | - | 1.5 | R | 12 | R | 0.25 | S |
| 295 | 2019 | M | 33 | 0.5 | I | - | - | 2 | R | 0.75 | I | 0.5 | S |
| 296 | 2019 | M | 31 | 0.25 | I | - | - | 1.5 | R | 0.38 | I | 0.25 | S |
| 297 | 2019 | M | 31 | 6 | R | - | - | 0.5 | I | 16 | R | 0.032 | S |
| 298 | 2019 | M | 26 | - | - | - | - | 1 | R | 6 | R | 0.125 | S |
| 299 | 2019 | M | 23 | 0.19 | I | 0.012 | S | 32 | R | - | - | 0.5 | S |
| 300 | 2019 | M | 29 | 0.25 | I | 0.25 | S | 4 | R | 0.5 | I | 0.38 | S |
| 301 | 2019 | M | 21 | 0.094 | I | 0.002 | S | - | - | - | - | 0.125 | S |
| 302 | 2019 | F | 37 | 0.75 | I | - | - | 6 | R | 1.5 | R | 0.5 | S |
| 303 | 2019 | M | 35 | - | - | - | - | 0.75 | R | 12 | R | 0.125 | S |
| 304 | 2019 | M | 37 | - | - | - | - | 3 | R | 0.25 | S | 0.25 | S |
| 305 | 2019 | M | 21 | 0.25 | I | 0.006 | S | - | - | - | - | 0.75 | S |
| 306 | 2019 | M | 20 | - | - | - | - | 0.004 | S | 0.25 | S | 8 | R |
| 307 | 2019 | M | 20 | 256 | R | 0.008 | S | - | - | - | - | 0.75 | S |
| 308 | 2019 | M | 20 | 0.19 | I | 0.006 | S | - | - | 0.5 | I | 0.75 | S |
| 309 | 2019 | M | 21 | 0.064 | S | 0.002 | S | - | - | - | - | 0.064 | S |
| 310 | 2019 | M | 29 | 0.064 | S | 0.012 | S | - | - | 0.5 | I | 0.75 | S |
| 311 | 2019 | M | 25 | 0.094 | I | 0.002 | S | - | - | 16 | R | 0.19 | S |
| 312 | 2019 | M | 28 | 0.19 | I | 0.032 | S | - | - | - | - | 256 | R |
| 313 | 2019 | M | 32 | 256 | R | 0.002 | S | - | - | - | - | 0.125 | S |
| 314 | 2019 | M | 19 | 0.094 | I | 0.002 | S | - | - | - | - | 0.38 | S |
| 315 | 2019 | M | 22 | 0.064 | S | 0.002 | S | - | - | - | - | 0.5 | S |
| 316 | 2019 | M | 18 | 0.023 | S | 0.002 | S | - | - | - | - | 0.064 | S |
| 317 | 2019 | M | 19 | 0.016 | S | 0.002 | S | - | - | - | - | 0.016 | S |
| 318 | 2019 | M | 26 | 3 | R | 0.064 | S | - | - | - | - | 0.38 | S |
| 319 | 2019 | M | 19 | 0.25 | I | 0.004 | S | - | - | - | - | 0.38 | S |
| 320 | 2019 | M | 28 | 0.094 | I | 0.006 | S | - | - | - | - | 0.38 | S |
| 321 | 2019 | M | 15 | 256 | R | 0.003 | S | - | - | - | - | 0.38 | S |
| 322 | 2019 | M | 35 | 0.19 | I | 0.002 | S | - | - | - | - | 0.38 | S |
| 323 | 2019 | F | 26 | 0.064 | S | 0.002 | S | - | - | - | - | 0.19 | S |
| 324 | 2019 | F | 36 | 8 | R | 0.002 | S | - | - | - | - | 0.125 | S |
| 325 | 2019 | M | 24 | 0.064 | S | 0.016 | S | - | - | - | - | 0.25 | S |
| 326 | 2019 | M | 24 | 0.125 | I | 0.004 | S | - | - | - | - | 0.25 | S |
| 327 | 2019 | M | 21 | 0.125 | I | 0.002 | S | - | - | - | - | 0.19 | S |
| 328 | 2019 | M | 22 | 24 | R | 0.002 | S | - | - | - | - | 0.125 | S |
| 329 | 2019 | M | 22 | 0.094 | I | 0.023 | S | - | - | - | - | 0.125 | S |
| 330 | 2019 | M | 36 | 0.125 | I | 0.002 | S | - | - | - | - | 0.125 | S |
| 331 | 2019 | M | 30 | 0.38 | I | 0.016 | S | - | - | - | - | 0.75 | S |
| 332 | 2019 | M | 26 | 256 | R | 0.002 | S | 0.75 | R | - | - | 0.125 | S |
| 333 | 2019 | M | 31 | 1 | I | 0.023 | S | 12 | R | - | - | 2 | R |
| 334 | 2019 | M | 25 | 0.25 | I | 0.012 | S | - | - | - | - | 0.75 | S |
| 335 | 2019 | F | 24 | 32 | R | 0.002 | S | - | - | - | - | - | - |
| 336 | 2019 | M | 20 | 3 | R | 0.002 | S | - | - | - | - | 0.047 | S |
| 337 | 2019 | M | 21 | 0.19 | I | 0.006 | S | 1.5 | R | - | - | 0.5 | S |
| 338 | 2019 | M | 23 | 0.25 | I | 0.008 | S | - | - | - | - | 0.75 | S |
| 339 | 2019 | M | 21 | 48 | R | 0.002 | S | 1 | R | - | - | 0.125 | S |
| 340 | 2019 | M | 27 | 1 | I | 0.002 | S | 0.5 | I | - | - | 0.19 | S |
| 341 | 2019 | M | 32 | 0.5 | I | 0.002 | S | 0.75 | R | - | - | 0.064 | S |
| 342 | 2019 | M | 20 | 0.19 | I | 0.004 | S | 1.5 | R | - | - | 0.38 | S |
| 343 | 2019 | M | 19 | 6 | R | 0.002 | S | 0.75 | R | - | - | 0.094 | S |
| 344 | 2019 | M | 24 | 0.25 | I | 0.016 | S | 1.5 | R | - | - | 0.5 | S |
| 345 | 2019 | M | 25 | 0.064 | S | 0.004 | S | 6 | R | - | - | 0.094 | S |
| 346 | 2019 | M | 27 | 3 | R | 0.002 | S | 0.25 | I | - | - | 0.094 | S |
| 347 | 2019 | M | 40 | 4 | R | 0.004 | S | 0.75 | R | - | - | 0.38 | S |
| 348 | 2019 | M | 21 | 0.75 | I | 0.006 | S | - | - | - | - | 256 | R |
| 349 | 2019 | M | 29 | 6 | R | 0.002 | S | 0.38 | I | - | - | 0.125 | S |
| 350 | 2019 | M | 27 | 0.38 | I | 0.002 | S | 0.064 | S | - | - | 0.094 | S |
| 351 | 2019 | M | 21 | 8 | R | 0.002 | S | 0.75 | R | - | - | 0.125 | S |
| 352 | 2019 | M | 26 | 0.75 | I | 0.002 | S | 4 | R | - | - | - | - |
| 353 | 2019 | M | 34 | 0.19 | I | 0.004 | S | 1.5 | R | - | - | 0.38 | S |
| 354 | 2019 | M | 34 | 0.094 | I | 0.002 | S | 1 | R | - | - | 0.125 | S |
| 355 | 2019 | M | 37 | 0.5 | I | 0.012 | S | 0.75 | R | - | - | 0.38 | S |
| 356 | 2019 | M | 60 | 24 | R | 0.004 | S | 1.5 | R | 24 | R | 0.19 | S |
| 357 | 2019 | F | 32 | 0.75 | I | 0.002 | S | 0.25 | I | - | - | 0.5 | S |
| 358 | 2019 | M | 32 | 0.094 | I | 0.002 | S | 0.19 | I | - | - | 0.19 | S |
| 359 | 2019 | M | 17 | 0.25 | I | 0.002 | S | 0.008 | S | - | - | 3 | R |
| 360 | 2019 | M | 23 | 4 | R | 0.003 | S | 0.75 | R | 0.5 | I | 0.19 | S |
| 361 | 2019 | M | 22 | 3 | R | 0.003 | S | 1 | R | 6 | R | 0.25 | S |
| 362 | 2019 | M | 29 | 0.75 | I | 0.008 | S | 1 | R | 0.75 | I | 1 | S |
| 363 | 2019 | M | 36 | 3 | R | 0.004 | S | 2 | R | 16 | R | 0.38 | S |
| 364 | 2019 | M | 17 | 0.016 | S | 0.002 | S | 1 | R | 0.19 | S | 0.38 | S |
| 365 | 2019 | M | 25 | 0.38 | I | 0.012 | S | 3 | R | 1 | I | 1 | S |
| 366 | 2019 | M | 20 | 6 | R | 0.002 | S | 0.25 | I | 0.5 | I | 0.25 | S |
| 367 | 2019 | M | 23 | 1 | I | 0.002 | S | 0.023 | S | 0.125 | S | 0.016 | S |
| 368 | 2019 | M | 20 | 8 | R | 0.002 | S | 0.25 | I | 0.5 | I | 0.19 | S |
| 369 | 2019 | M | 24 | 0.125 | I | 0.008 | S | 2 | R | 0.75 | I | 0.5 | S |
| 370 | 2019 | M | 34 | 0.75 | I | 0.002 | S | 0.25 | I | 24 | R | 0.19 | S |
| 371 | 2019 | M | 19 | 0.75 | I | 0.002 | S | 1 | R | 16 | R | 0.25 | S |
| 372 | 2019 | M | 20 | 2 | R | 0.003 | S | 0.25 | I | 32 | R | 0.38 | S |
| 373 | 2019 | M | 18 | 0.25 | I | 0.012 | S | 1.5 | R | 0.75 | I | 0.5 | S |
| 374 | 2019 | M | 22 | 0.094 | I | 0.002 | S | 0.125 | I | 4 | R | 0.19 | S |
| 375 | 2019 | M | 37 | 0.016 | S | 0.002 | S | 3 | R | 0.25 | S | 1 | S |
| 376 | 2019 | M | 21 | 0.094 | I | 0.002 | S | 0.5 | I | 8 | R | 0.125 | S |
| 377 | 2019 | M | 26 | 12 | R | 0.002 | S | 1 | R | 0.75 | I | 0.125 | S |
| 378 | 2019 | M | 42 | 0.016 | S | 0.002 | S | 2 | R | 0.38 | I | 0.75 | S |
| 379 | 2019 | M | 33 | 2 | R | 0.002 | S | 1 | R | 32 | R | 0.19 | S |
| 380 | 2019 | M | 20 | 0.064 | S | 0.002 | S | 0.002 | S | 0.5 | I | 0.5 | S |
| 381 | 2019 | M | 50 | 0.19 | I | 0.012 | S | 4 | R | 0.75 | I | 0.75 | S |
| 382 | 2019 | M | 26 | 0.125 | I | 0.002 | S | 1 | R | 16 | R | 0.25 | S |
| 383 | 2019 | M | 18 | 48 | R | 0.003 | S | 1.5 | R | 24 | R | 0.38 | S |
| 384 | 2019 | M | 38 | 256 | R | 0.003 | S | 1 | R | 1 | I | 0.25 | S |
| 385 | 2019 | M | 18 | 12 | R | 0.004 | S | 3 | R | 24 | R | 0.5 | S |
| 386 | 2019 | M | 42 | 0.19 | I | 0.004 | S | 0.5 | I | 16 | R | 1 | S |
| 387 | 2019 | M | 20 | 1.5 | R | 0.002 | S | 0.5 | I | 12 | R | 0.5 | S |
| 388 | 2020 | M | 22 | 0.25 | I | 0.003 | S | - | - | 0.38 | I | 0.75 | S |
| 389 | 2020 | M | 35 | 0.047 | S | 0.002 | S | 0.002 | S | 0.125 | S | 0.094 | S |
| 390 | 2020 | M | 20 | 0.5 | I | 0.012 | S | - | - | 0.75 | I | 1 | S |
| 391 | 2020 | M | 32 | 0.38 | I | 0.003 | S | - | - | 12 | R | 0.5 | S |
| 392 | 2020 | M | 26 | 32 | R | 0.002 | S | 0.25 | I | 8 | R | 0.064 | S |
| 393 | 2020 | M | 18 | 32 | R | 0.002 | S | 0.5 | I | 8 | R | 0.25 | S |
| 394 | 2020 | M | 31 | 0.125 | I | 0.002 | S | 0.5 | I | 0.38 | I | 0.25 | S |
| 395 | 2020 | M | 24 | 0.19 | I | 0.003 | S | 0.006 | S | 0.38 | I | 2 | R |
| 396 | 2020 | M | 20 | 32 | R | 0.004 | S | - | - | 16 | R | 0.38 | S |
| 397 | 2020 | M | 31 | 0.125 | I | 0.002 | S | 0.25 | I | 8 | R | 0.5 | S |
| 398 | 2020 | M | 32 | 0.5 | I | 0.002 | S | - | - | 12 | R | 0.19 | S |
| 399 | 2020 | M | 24 | 0.38 | I | 0.006 | S | 1.5 | R | 0.5 | I | 1 | S |
| 400 | 2020 | M | 34 | 2 | R | 0.003 | S | 0.25 | I | 2 | R | 0.094 | S |
| 401 | 2020 | M | 18 | 0.25 | I | 0.016 | S | 2 | R | 1 | I | 1 | S |
| 402 | 2020 | M | 40 | 0.125 | I | 0.008 | S | 0.002 | S | 0.125 | S | 0.75 | S |
| 403 | 2020 | M | 44 | 0.016 | S | 0.002 | S | 0.002 | S | 0.25 | S | 0.75 | S |
| 404 | 2020 | M | 17 | 0.75 | I | 0.002 | S | 0.5 | I | 16 | R | 0.25 | S |
| 405 | 2020 | M | 26 | 0.016 | S | 0.002 | S | 0.002 | S | 0.094 | S | 0.094 | S |
| 406 | 2020 | M | 24 | 0.5 | I | 0.003 | S | 1 | R | 1.5 | R | 0.25 | S |
| 407 | 2020 | M | 50 | 0.25 | I | 0.006 | S | 0.125 | I | 4 | R | 0.5 | S |
| 408 | 2020 | M | 20 | 3 | R | 0.003 | S | 1 | R | 12 | R | 0.25 | S |
| 409 | 2020 | M | 35 | 6 | R | 0.002 | S | 0.75 | R | 0.75 | I | 0.094 | S |
| 410 | 2020 | M | 44 | 0.125 | I | 0.002 | S | 0.75 | R | 0.38 | I | 0.25 | S |
| 411 | 2020 | M | 38 | 0.125 | I | 0.002 | S | 0.004 | S | 0.75 | I | 1 | S |
| 412 | 2020 | M | 23 | 0.016 | S | 0.002 | S | 0.002 | S | 0.5 | I | 0.5 | S |
| 413 | 2020 | M | 21 | 1 | I | 0.19 | S | 6 | R | 0.5 | I | 256 | R |
| 414 | 2020 | M | 25 | 0.38 | I | 0.008 | S | 4 | R | 0.5 | I | 0.75 | S |
| 415 | 2020 | M | 23 | 0.25 | I | 0.012 | S | 3 | R | 1 | I | 1 | S |
| 416 | 2020 | M | 27 | 0.047 | S | 0.012 | S | 3 | R | 0.25 | S | 0.125 | S |
| 417 | 2020 | M | 27 | 6 | R | 0.002 | S | 0.5 | I | 0.5 | I | 0.047 | S |
| 418 | 2020 | M | 16 | 0.25 | I | 0.002 | S | 0.19 | I | 16 | R | 0.064 | S |
| 419 | 2020 | M | 20 | 0.064 | S | 0.002 | S | 0.75 | R | 0.25 | S | 0.19 | S |
| 420 | 2020 | M | 46 | 0.064 | S | 0.002 | S | 0.023 | S | 2 | R | 0.125 | S |
| 421 | 2020 | M | 22 | 0.094 | I | 0.003 | S | 1 | R | 3 | R | 0.047 | S |
| 422 | 2020 | M | 28 | 32 | R | 0.032 | S | 3 | R | 16 | R | 0.094 | S |
| 423 | 2020 | M | 58 | 2 | R | 0.002 | S | 0.5 | I | 0.25 | S | 0.016 | S |
| 424 | 2020 | M | 31 | 0.125 | I | 0.004 | S | 6 | R | 0.25 | S | 0.064 | S |
| 425 | 2020 | M | 28 | 12 | R | 0.004 | S | 1 | R | 12 | R | 0.125 | S |
| 426 | 2020 | M | 35 | 1 | I | 0.006 | S | 0.5 | I | 0.25 | S | 0.125 | S |
| 427 | 2020 | M | 21 | 0.064 | S | 0.002 | S | 0.5 | I | 0.5 | I | 0.19 | S |
| 428 | 2020 | M | 45 | 3 | R | 0.003 | S | 1.5 | R | 6 | R | 0.125 | S |
| 429 | 2020 | M | 32 | 0.064 | S | 0.002 | S | 2 | R | 0.25 | S | 0.064 | S |
| 430 | 2020 | M | 27 | 2 | R | 0.002 | S | 0.25 | I | 0.032 | S | 0.032 | S |
| 431 | 2020 | M | 23 | 4 | R | 0.008 | S | 0.38 | I | 6 | R | 0.25 | S |
| 432 | 2020 | M | 34 | 0.75 | I | 0.002 | S | 0.25 | I | 8 | R | 0.125 | S |
| 433 | 2020 | M | 26 | 0.125 | I | 0.004 | S | 2 | R | 0.5 | I | 0.25 | S |

F, female; I, intermediate; M, male; -, not tested; R, resistance; S, susceptibility.

^*^The specimen was not viable for confirmatory Etest. Accordingly, the susceptibility characterisation was based on whole-genome sequencing and a lack of all known genetic resistance determinants for ceftriaxone in the genome sequence.
